# Outpatient parenteral antibiotic treatment for infective endocarditis: A retrospective observational evaluation

**DOI:** 10.1016/j.clinme.2024.100213

**Published:** 2024-04-21

**Authors:** Dr Ralph Schwiebert, Dr Sokolayam Atanze, Dr Uchechika Iroegbu, Dr Molly Wilkins, Dr Jonathan A T Sandoe

**Affiliations:** a Department of Microbiology, Leeds Teaching Hospitals NHS Trust, Leeds, England, United Kingdom; b Department of Anaesthesia and Intensive Care Medicine, Countess of Chester Hospital, Chester, England, United Kingdom; c Leeds Institute of Medical Research, University of Leeds, Leeds, England, United Kingdom

**Keywords:** Outpatient antibiotic therapy, Infective endocarditis

## Abstract

**Background:**

Infective endocarditis (IE) requires long courses of intravenous (IV) antibiotics. Outpatient parenteral antibiotic therapy (OPAT) saves resources, improves the patient experience and allows care in their preferred place; however, questions remain about safety when treating IE patients. This study evaluates OPAT management of IE patients in our region between 2006 and 2019.

**Methods:**

This is a retrospective observational evaluation and description of outcomes and adherence to suitability criteria, according to British Society for Antimicrobial Chemotherapy (BSAC) guidelines.

**Results:**

We identified five models of OPAT delivery. The number of patients treated expanded significantly over time. Of 101 patients, six (6%) suffered poor outcomes, but each patient had contributing factors outside of the primary infection. Median OPAT duration was 12 days and 1,489 hospital bed days were saved.

**Conclusions:**

In a setting where there was good adherence to BSAC criteria, treating IE patients using OPAT services was safe. Complications observed were likely independent of treatment location. Significant bed days were saved.

**Summary box**

What is known?

Infective endocarditis remains a challenging condition to treat. OPAT is an attractive option as it can save hospital bed days and improve the patient experience.

What is the question?

There are potential severe adverse outcomes for undertreated patients, which may be more common if patients not suitable for OPAT are treated on an outpatient basis.

What was found?

Our study demonstrates that OPAT is a feasible and effective option for treating IE in a broad range of patients and provides good results in terms of treatment failure, readmission, death and relapse. It also highlights the heterogeneity of patients and OPAT services.

How might this impact on clinical practice?

This study contributes to the growing evidence that supports the use of OPAT for IE, particularly where it is delivered through a dedicated community intravenous administration team.

Alt-text: Unlabelled box

## Introduction

Infective endocarditis (IE) is an uncommon condition affecting the endocardium and endocardial devices.^1^ The incidence of IE in England has recently been reported as 42–68 per million people per month and is increasing.^2^ Although uncommon, IE is important because it results in significant morbidity and mortality, with an inpatient mortality rate of 20–30%.^3^^,^^4^ IE is notoriously difficult to treat and frequently requires prolonged hospitalisation.^1^ A cornerstone of treatment is intravenous antimicrobial therapy, which is generally required for 4 to 6 weeks.^5^ Hospital stays carry their own risks including healthcare associated infections, which are a particular threat for older and frail patients who now comprise a large proportion of those suffering from IE in many counties.^6^^,^^7^

Outpatient parenteral antimicrobial therapy (OPAT), is a way of delivering antibiotic treatment to patients without the need to stay in hospital. It offers cost savings, allows greater inpatient capacity due to bed days saved and improves patient satisfaction.^8^ Despite these benefits there are risks, including adverse drug reactions and IV access complications, as well as unexpected changes in the patient's condition.^9^ Intravascular catheter-related bloodstream infection is a particular concern in IE patients as it appears to increase the risk of mortality.^10^

For patients with IE to be considered for OPAT, the British Society for Antimicrobial Chemotherapy (BSAC) guidelines stipulate that they must satisfy general suitability criteria as well as condition-specific requirements.^5^^,^^6^ A number of studies have reviewed the safety and effectiveness of OPAT for the treatment of IE and although they have concluded that it is safe, high readmission rates (4–33%) and mortality (0–5.5%) have been reported, raising questions about the patient selection criteria.^11^, ^12^, ^13^, ^14^, ^15^ In addition, previous studies have generally been evaluated from an OPAT service provider perspective, potentially biasing findings. Because IE is a condition fraught with high morbidity and mortality and because readmission may undo much of the economic case for OPAT, as well as the patient satisfaction benefits, we aimed to audit compliance with BSAC recommendations for suitability for OPAT of IE. We also sought to describe models and trends in the utilisation of OPAT and to evaluate current outcomes from the perspective of patients with the condition, i.e. not limited to formal OPAT service referral

## Methods

### Ethics

This study was categorised as an audit and service evaluation and did not therefore require ethical approval. Data governance was managed according to Caldicott principles.

### Study design

This was a retrospective, descriptive, service evaluation, including audit compliance with BSAC recommendation 5.12 concerning suitability for OPAT treatment of IE and treatment outcome data.^5^ For all patients treated with OPAT, the percentage who were fully compliant with each requirement was assessed: 1) stable and responding well to therapy; 2) without signs of heart failure; 3) without any of the indications for urgent/emergency cardiac surgery; 4) without uncontrolled extra-cardiac foci of infection. Where non-compliance was identified, the reasons were explored.

Indications for surgery were considered to be: uncontrolled heart failure, uncontrolled cardiac focus of infection (defined as locally uncontrolled infection, including abscess, false aneurysm, enlarging vegetation, persisting fever and positive blood culture for ≥10 days after commencing appropriate antimicrobial therapy) and major embolic risk. Uncontrolled extracardiac foci of infection included: 1) undrained brain abscesses that were considered to need surgical drainage by a neurosurgeon 2) lung abscess/embolic pulmonary infection 3) any undrained splenic abscess 3) vertebral osteomyelitis with or without abscess considered to need surgical intervention by a spinal surgeon 4) septic arthritis without washout and ongoing symptoms of infection.

Outcomes measures were modified from previous.^11^ The primary outcome ‘OPAT failure’ was defined as unplanned readmission or death during OPAT. Secondary outcomes were: (i) relapse of endocarditis (ii) the need for emergency cardiac surgery during OPAT (iii) development of antibiotic resistance during OPAT or relapses, and (iv) intravascular catheter-related bloodstream infection during OPAT. Relapse was defined as an initial response to therapy followed by reappearance of clinical illness fulfilling diagnostic criteria for IE and cause by the same pathogen as the index episode within a year following completion of therapy. Data on 1-year all-cause mortality was collected to compare to previous studies. As patients with endocarditis often have other significant co-morbidities, we separately reported the percentage of patients that had no microbiological evidence of relapse within 1 year of cessation of treatment – this is the outcome that best aligns with microbiological cure.

We give a descriptive account of OPAT services, the models of delivery used, discussion of outcomes, and estimate of bed days saved.

### Setting

This study was conducted in hospitals within the Leeds Teaching Hospitals NHS Trust (LTHT) with over 2,500 inpatient beds and receives tertiary referrals from a population of over two million. OPAT services were provided by LTHT as well as five referring local hospitals.

### Participants

Inclusion criteria: All adult patients managed by the Leeds endocarditis service between 1 January 2006 and 31 December 2019 who fulfilled criteria for definite or possible IE according to modified Duke's criteria and had at least 1 day of intravenous therapy for IE while not an inpatient were eligible.^16^ Patients were followed up regardless of which regional service administered OPAT therapy after completing inpatient treatment at our tertiary referral centre. Analysis was stopped at the end of December 2019 because of the dramatic, abnormal effect that COVID-19 had on service provision. Patients fulfilling inclusion criteria were identified by using an in-house Leeds Infective Endocarditis Service electronic database. Data collection for this database started prospectively in 2004. Consecutive patients fulfilling the inclusion criteria were included to reduce the risk of bias and any reasons for exclusion described. Patient selection was carried out without knowledge of outcomes.

### Other variables

Other variables collected included: age, sex, year of admission, microbiological cause of IE, valve and valve type affected, injection of intravenous drug in the 3 months prior to admission, OPAT antimicrobial regimen, duration of inpatient antibiotic therapy and duration of OPAT therapy and outcomes.

Day 1 of therapy was considered to be the first day of appropriate antibiotics commenced for treatment of IE. Day 1 of OPAT was the first day that intravenous antibiotics were administered outside the inpatient setting.

### Statistical methods

Microsoft Office Excel 2016 was used to collect data and calculate means, medians and ranges. Median values and ranges were calculated for non-normally distributed continuous variables. Means were calculated for normally distributed continuous variables. Risk ratios and their statistical significance were calculated using MedCalc.^17^ The Kaplan-Meier curves in Fig. 2 were generated using Stata Statistical Software Release 18 (StataCorp, College Station, Texas, USA).

## Results

**Fig. 1:**
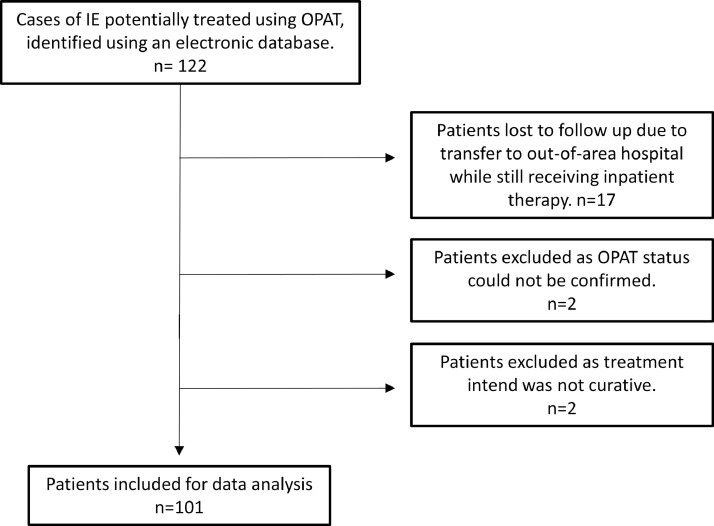
Flow diagram of cases identified using an electronic database and subsequent including and exclusion.

An initial search identified 122 patients who satisfied the eligibility criteria of this study, of which 101 were ultimately included for analysis. See Fig. 1 for details on case exclusions. Eighty-three patients received OPAT via Leeds Teaching Hospitals NHS Trust, and 18 patients received OPAT via their local referring hospital. During the same time period 1,396 patients were treated for endocarditis on an inpatient basis at Leeds Teaching Hospitals Trust.

### Patient and treatment characteristics

The basic characteristics of the 101 included patients were very similar to the total population of patients seen by our IE service (regardless of inpatient or outpatient therapy), including age, gender, microbiological causes, type of IE and cardiac structures affected (previously reported)^18^ and is largely in line with other European epidemiological data for IE (Table 1).^19^ We included two persons who inject intravenous drugs. While this is not our usual practice, both were deemed suitable on individual review.

The duration of outpatient therapy ranged from 1 to 59 days, with a median of 12 days. For three patients the OPAT treatment duration could not be determined. For the remaining 98 patients, a total of 1,489 days of hospital treatment were saved (Table 2).

**Table 1:** Baseline characteristics of patients treated for infective endocarditis using outpatient parenteral antibiotic therapy (OPAT).

**Number of patients**	101
**Age** (years, median)	68 (range 18–92)
**Gender**	
Male	70 (69%)
Female	31 (31%)
**Person who injects drugs**	2 (2%)
**Type of IE**	
Native valve	63 (62%)
Prosthetic valve	28 (28%)
Implantable Cardiac Electronic Device (ICED)	8 (8%)
Other	2 (2%)
**Cardiac structure affected**	
Aortic valve	41 (41%)
Mitral valve	29 (29%)
Tricuspid valve	10 (10%)
Pulmonary valve	2 (2%)
Other (multiple valves, non-valvular prosthetic structures, miscellaneous non-valvular native structures)	19 (19%)
**Microbiological cause of IE**	
Streptococci - Other (primarily oral streptococci)	41 (41%)
Staphylococcus aureus	17 (17%)
Coagulase Negative Staph	16 (16%)
Enterococci	10 (10%)
Other (HACEK, gram negative rods, fungal)	8 (8%) (3 *P. aeruginosa, 1 C.parapsilosis*, 1 *Leptotrichia*, 1 *P. acnes, 2 multiple bacterial pathogens*)
No pathogen identified	6 (6%)
Beta-haemolytic streptococci	3 (3%)

Data recorded as number of patients (with percentages in brackets), unless otherwise specified.

**Table 2:** Characteristic of OPAT episodes and services models.

OPAT Antibiotic used	
Teicoplanin	51 (50%) • 48 IV Teicoplanin only • 2 +PO rifampicin • 1 +PO rifampicin + ciprofloxacin
Vancomycin	8 (8%) 6 IV vancomycin only 1 +IV gentamicin +PO rifampicin 1 +PO rifampicin
Flucloxacillin	4 (4%) • 3 IV flucloxacillin only • 1 +PO rifampicin
Daptomycin	26 (26%) • 10 IV daptomycin only, 13 +PO rifampicin • 1 +PO ciprofloxacin • 1 +PO linezolid • 1 +PO linezolid + rifampicin
Ceftriaxone	5 (5%)
Other	7 (7%) • 1 ceftriaxone + teicoplanin • 1 ciprofloxacin • 1 anidulafungin • 2 ceftolozane-tazobactam • 2 benzylpenicillin

**Antibiotic therapy duration**	
Duration of inpatient antibiotic therapy (days, median)	27 (range 6 to 94)
Duration of outpatient antibiotic therapy (days, median)	12 (range 1 to 59)
**OPAT models used**	
Community intravenous antibiotic service (CIVAS)	63 (62%)
Ward attender	21 (21%)
Dialysis	11 (11%)
Unknown	3 (3%)
Clinic attender	2 (2%)
Self OPAT	1 (1%)

Data recorded as number of patients (with percentages in brackets), unless otherwise specified. PO, per oral; IV, intravenous

The five OPAT service models used included: community intravenous antibiotic services (CIVAS), ward attenders, clinic attenders, self-OPAT and dialysis services for patients that already required renal replacement therapy. More than half of all patients (62%) were treated using the CIVAS service, which is a dedicated team run by nurses, doctors and pharmacists that administer antibiotics at a patient's place of residence. The proportion of patients that attended a hospital ward for outpatient antibiotic treatment fell during the study period from 50% during the first half of the study period to 16% during the second half. Over the same time interval, the total number of patients treated for IE using OPAT increased by a factor of four. Due to their once-a-day dosing schedule, daptomycin and teicoplanin accounted for over three quarters of all OPAT prescriptions.

### Compliance with BSAC recommendation for OPAT suitability

There was high adherence to recommendations for the selection patients for OPAT therapy (Table 3). However, there was weaker adherence to the recommendation for daily monitoring (88%). In total there were 15 patients in whom BSAC recommendations were not adhered to (three of whom scored in two separate domains for a total of 18 instances of non-adherence). Reasons for non-adherence to the recommendations were as follows:

- 11 patients with renal replacement therapy requirements managed their antibiotic administration through their intermittent haemodialysis service, which does not monitor patients on a daily basis. One patient was administered IV antibiotics in their own home with help of their trained family members.
- All three patients that were not stable/responding to therapy prior to discharge were seeking self-discharge against medical advice and OPAT was offered as a compromise. One patient had a negative outcome, suffering a stroke and requiring readmission while still on IV antibiotics. This patient was also on haemodialysis.
- One patient was discharged with heart failure as his condition was as optimised as possible and was approaching end of life care. No adverse outcome was reported.
- One patient was discharged prior to receiving indicated valve surgery as he had multiple comorbidities and determining the timing of surgery was a difficult multi-disciplinary team decision. No adverse outcome was reported.
- One patient was discharged with an uncontrolled focus of infection (neck abscess) which the patient declined to have surgically drained. No adverse outcome reported.

**Table 3:** Compliance with BSAC recommendation for suitability on starting OPAT.

Stable and responding to therapy prior to OPAT	97% (98 of 101)
No signs of heart failure	99% (99 of 100)
No indication for surgery prior to discharge	99% (100 of 101)
No uncontrolled extra-cardiac foci of infection	99% (100 of 101)
System in place for daily monitoring	88% (88/100)

Data displayed as percentage of case adhering to recommendations, with count numbers in brackets. A denominator less than 101 reflects cases of missing data.

**Table 4:** Adverse treatment outcomes of patients treated with OPAT for infective endocarditis.

OPAT failure (death or unplanned readmission during OPAT)	3% (3 of 99)
Relapse of endocarditis (within 1 year of completion of antibiotics)	3% (3 of 99)
Need for emergency cardiac surgery	0% (0 of 99)
Catheter related bloodstream infection	0%` (0 of 98)

Data displayed as percentage of cases, with count numbers in brackets. A denominator less than 101 reflects cases of missing data.

ESRF, end stage renal failure; MRSA, methicillin-resistant *Staphylococcus aureus*; MSSA, methicillin-susceptible *Staphylococcus aureus*.

### Adverse treatment outcomes

**Fig. 2:**
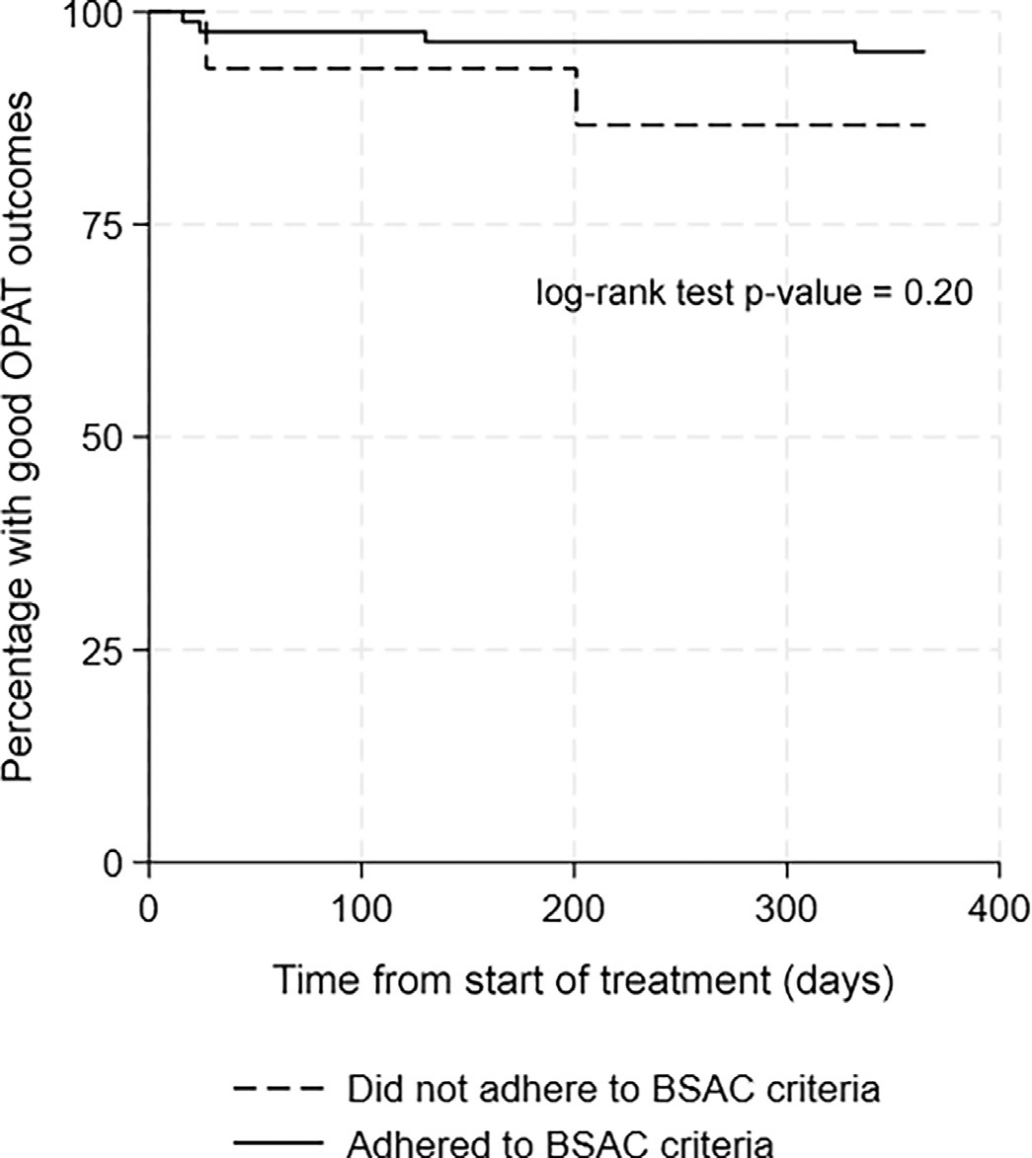
Kaplan–Meier curve of the percentage of patients being complication free over time. There were two negative outcomes in the group of 15 patients that did not adhere to BSAC criteria for OPAT (one readmission, one relapse) and four negative outcomes in the group of 86 patients that did adhere to BSAC criteria (two relapses, two deaths). The difference between groups is non-significant at *p =* 0.20 (log-rank test).

In total, there were six negative treatment outcomes (Table 4) and details of each case are summarised in Table 5. Two occurred in the 15 patients in whom BSAC recommendations for OPAT suitability were not strictly adhered to (one stroke, one relapse). The remaining four negative outcomes were distributed among the remaining 86 patients. While the relative risk of developing a negative outcome was higher in the group that did not follow BSAC recommendations for OPAT, it did not reach statistical significance (risk ratio 2.87, *p* = 0.20). A time-to-negative-event Kaplan-Meier curve comparing those who adhered to BSAC criteria vs those that did not is shown in Fig. 2. There was no statistically significant difference in the occurrence of negative outcomes between the two groups (log-rank test, *p =* 0.20).

**Table 5:** Details of all cases of OPAT treatment failures during the study period.

Patient	Adverse outcome	Adherence to suitability criteria	Type of OPAT	Type of valve	Organism	Contributing factors
1	Death	Yes	CIVAS	Native tricuspid valve	*E. faecalis*	Elderly, with metastatic cancer under palliative care.
3	Death	Yes	Ward attender	Native mitral valve	MRSA	Advanced metastatic breast ca with decompensated liver failure.
3	Readmission (stroke)	No - not stable at discharge - not monitored daily (haemodyalisis)	Haemodialysis unit	Prosthetic aortic valve	MSSA	ESRF. Self-discharge against medical advice.
4	Relapse	Yes	CIVAS	Native aortic valve	*C. parapsilosis*	Fungal IE is difficult to treat and prone to relapse
5	Relapse	No -not monitored daily (haemodialysis)	Haemodialysis unit	Native mitral valve	MSSA	ESRF, complex multi focal infection.
6	Relapse	Yes	CIVAS	ICED (device removed)	*S. sanguinis*	Sarcoidosis on high dose steroids

ESRF, end stage renal failure; MRSA, methicillin-resistant *Staphylococcus aureus*; MSSA, methicillin-susceptible *Staphylococcus aureus*.

Likewise, the difference in relative risk of developing complications in a dedicated CIVAS setting vs the more ad-hoc alternatives also did not reach statistical significance (risk ratio = 1.65, *p =* 0.52).

Of the three relapses, one patient was on dialysis and the infection was with *S aureus. S aureus* infections are common in this patient population and it is thus difficult to ascertain if it is truly a relapse of a reinfection. Overall, 97% of patients had no evidence of microbiological relapse within 1 year of cessation of therapy.

Sixteen patients died within 1 year of diagnosis, resulting in a 16% 1-year all-cause mortality. None had microbiological evidence of relapse. We note that three patients had advanced cancer during their treatment (two of which died during therapy and one shortly after), three were on dialysis, one was a complex surgical case and one received OPAT in order to deliver patient centred care, despite not being best medical practice. Patients from these cohorts are often excluded from traditional OPAT studies.

### Non-curative intent

Two patients were excluded from analysis as their treatment outcome was not curative. Both had complicated prosthetic valve endocarditis and could not undergo the required surgery.

## Discussion

The review of our tertiary infective endocarditis service highlights that the overwhelming majority of patients treated using OPAT met BSAC suitability criteria and were safely treated.

Previous OPAT studies have reported a relapse rate between 1 and 5.5%, with another UK based study also citing a relapse rate and mortality of 3%, which is identical to our findings.^12^^,^^20^ The readmission rate during OPAT of 1% was significantly lower in our study compared to others who have previously reported it between 4 and 20% and may be due to diverging inclusion criteria and different thresholds for hospital readmission. The 1-year mortality rate reported here is higher than that cited for IE OPAT studies previously.^12^^,^^13^^,^^20^, ^21^, ^22^ However, studies of OPAT outcomes across different services are known to show significant variance due to the heterogeneity in services provision, inclusion criteria, patient populations and reporting standards.^23^ Our mortality data is in line with mortality data for IE cases overall, which is cited between 8 and 37%, and likely reflects that we treated a number of complex surgical cases, tenacious organisms, a significant number of patients on haemodialysis and patients near the end of their life for compassionate reasons.^24^, ^25^, ^26^, ^27^ Reassuringly, there was a low rate of microbiological relapse and conversely a high rate of microbiological cure.

We observed a change in how OPAT was delivered over time, with growing use of CIVAS and less *ad hoc* ward/clinic-based delivery. In line with national trends, we have seen an increasing uptake in OPAT services year on year.^23^ We note that there was less continuing oversight from infection specialists in the management of cases that were not treated by a structured CIVAS service and the suitability of this approach warrants further surveillance.

While patients that received non-CIVAS services did not have a statistically significant difference in outcomes, reporting of such incidents may be lower in a less formal OPAT setting. Justification for antibiotic duration was meticulously recorded for patients receiving CIVAS and there was regular clinical review and therapeutic drug monitoring, but this was much less rigorous for patients receiving non-CIVAS therapy. While the same monitoring may have occurred, it was often not documented. We encourage OPAT to be delivered through a CIVAS team as highlighted in national good practice guidelines.^6^

Concerning patients who had adverse outcomes, we recognise that most of them had non-infection factors that significantly contributed to their health and it is difficult to ascribe the occurrence of negative outcomes to OPAT therapy alone. The median duration of therapy for this population was high, but in part accounted for by a number of complex surgical cases, cases that could not undergo source control interventions due to frailty and cases where individuals refused best medical management.

### Strengths and limitations

This study differs from most previous studies of IE OPAT management in that we followed up all patients that were reviewed by our endocarditis team, regardless of which OPAT service ultimately treated them; in this respect we have reduced the risk of bias. We extended follow up to multiple surrounding referring hospitals that patients may have returned to. Nineteen patients which were treated as inpatients were transferred to hospitals that could not provide follow up data, but it is unlikely that a significant number of them will have received OPAT services.

We have endeavoured to minimise the risk of bias by collecting data from consecutive patients, but it is possible that we were unaware of a small number of IE patients that were treated via OPAT. We have not evaluated patients managed as inpatients to assess how many of them fulfilled BSAC criteria for OPAT, but were not discharged.

Numbers were small when doing subgroup analysis and while there was a trend to increased risk in patients treated by non-CIVAS models of OPAT delivery, this did not reach statistical significance. This was a retrospective analysis of all patients seen by our endocarditis team and was not powered towards a particular endpoint analysis.

## Conclusions

The use of OPAT for treating IE has increased substantially in our region during the study period and saved a significant number of bed days. The use of a formal CIVAS service increased significantly while the use of the informal ward attender model halved. We have concern over governance and antimicrobial stewardship with unstructured models of delivery. OPAT for IE was safe in settings where BSAC selection criteria were followed and our OPAT failure rates are in keeping with those reported elsewhere .

## Funding

No funders were involved in this study. RS was supported by a National Institute of Health Research academic clinical fellowship. In the last 5 years JS received research funding from MRC, EPSRC, NIHR, Welcome, Astellas, Pfizer, MSD, Lumos. JS has received funding for educational events from Tillotts Pharma and funding from Medtronic for research. SA, UI and MW have nothing to declare.

## Contributors

RS, SA, UI and JS developed the conceptual design of the study. RS, SA, UI, and MW collected clinical data. RS and SA performed data analysis and wrote the manuscript. JS UI and MW critically reviewed the manuscript.

## Declaration of competing interest

None of the authors have declared a conflict of interest.

## Appendix Supplementary materials

Image, application 1
