# Supplementary material for: Outpatient parenteral antibiotic treatment for infective endocarditis: A retrospective observational evaluation
Source: Clin Med (Lond). 2024 Apr 21;24(3):100213. doi: 10.1016/j.clinme.2024.100213 (PMC11101910; doi:10.1016/j.clinme.2024.100213)
Supplement: Supplementary file 1 [file mmc1.docx]

STROBE Statement—checklist of items that should be included in reports of observational studies

|  | Item No. | Recommendation | Page  No. | Relevant text from manuscript |
| --- | --- | --- | --- | --- |
| **Title and abstract** | 1 | (*a*) Indicate the study’s design with a commonly used term in the title or the abstract | 2 | “Retrospective observational evaluation” in abstract and similar in title. |
|  |  | (*b*) Provide in the abstract an informative and balanced summary of what was done and what was found | 2 | Abstract explains that this is a restrospective observational study with the outcome to evaluate patients against BSAC guidelines, describe outcomes and describe changes in OPAT over time. |
| Introduction | | | |  |
| Background/rationale | 2 | Explain the scientific background and rationale for the investigation being reported | 3 | Provided brief background to IE and OPAT and why it is valuable to evaluate the safety of OPAT in IE patients |
| Objectives | 3 | State specific objectives, including any prespecified hypotheses | 3-4 | Last two sentences in introduction lay out aims “we aim to….” |
| Methods | | | |  |
| Study design | 4 | Present key elements of study design early in the paper | 4-5 | 3 paragraphs explicitly on study design “retrospective, descriptive, service evaluation, including audit compliance with BSAC recommendation 5.12 concerning suitability for OPAT treatment of IE and treatment outcome data.” |
| Setting | 5 | Describe the setting, locations, and relevant dates, including periods of recruitment, exposure, follow-up, and data collection | 5 | Specific section on setting – tertiary teaching hospital and surrounding local hospitals that provided OPAT services. Dates and database/data collection stated in the “participants” section. |
| Participants | 6 | (*a*) *Cohort study*—Give the eligibility criteria, and the sources and methods of selection of participants. Describe methods of follow-up  *Case-control study*—Give the eligibility criteria, and the sources and methods of case ascertainment and control selection. Give the rationale for the choice of cases and controls  *Cross-sectional study*—Give the eligibility criteria, and the sources and methods of selection of participants | 5-6 | Eligibility/Inclusion criteria in “participants” on page 6 |
|  |  | (*b*) *Cohort study*—For matched studies, give matching criteria and number of exposed and unexposed  *Case-control study*—For matched studies, give matching criteria and the number of controls per case | - | Not applicable |
| Variables | 7 | Clearly define all outcomes, exposures, predictors, potential confounders, and effect modifiers. Give diagnostic criteria, if applicable | 4, 6 | Described in study design – BSAC criteria are the primary variable, other variables described on page 6 under “other variables” |
| Data sources/ measurement | 8* | For each variable of interest, give sources of data and details of methods of assessment (measurement). Describe comparability of assessment methods if there is more than one group | 4 | Clearly set out in “study design” |
| Bias | 9 | Describe any efforts to address potential sources of bias | 5, 15 | Described under “participants” how inclusion was handled to reduce bias and openly discussed weaknesses under “strengths and limitations” |
| Study size | 10 | Explain how the study size was arrived at | - | Not applicable |

Continued on next page

| Quantitative variables | 11 | Explain how quantitative variables were handled in the analyses. If applicable, describe which groupings were chosen and why | 6 | “Statistical methods” section. |
| --- | --- | --- | --- | --- |
| Statistical methods | 12 | (*a*) Describe all statistical methods, including those used to control for confounding | 6 | “Statistical methods” explains risk ration and p values. |
|  |  | (*b*) Describe any methods used to examine subgroups and interactions |  | Not applicable |
|  |  | (*c*) Explain how missing data were addressed |  | Not applicable |
|  |  | (*d*) *Cohort study*—If applicable, explain how loss to follow-up was addressed  *Case-control study*—If applicable, explain how matching of cases and controls was addressed  *Cross-sectional study*—If applicable, describe analytical methods taking account of sampling strategy | Fig 1 | Figure 1 shows how many patients could not be followed up. |
|  |  | (*e*) Describe any sensitivity analyses | - | Not applicable |
| Results | | | | |
| Participants | 13* | (a) Report numbers of individuals at each stage of study—eg numbers potentially eligible, examined for eligibility, confirmed eligible, included in the study, completing follow-up, and analysed | Fig 1 | Figure 1 shows how many patients could not be followed up. |
|  |  | (b) Give reasons for non-participation at each stage | Fig 1 | Yes |
|  |  | (c) Consider use of a flow diagram | Fig 1 | Yes, figure 1 |
| Descriptive data | 14* | (a) Give characteristics of study participants (eg demographic, clinical, social) and information on exposures and potential confounders | Table 1 | Table 1 “Baseline characteristics” |
|  |  | (b) Indicate number of participants with missing data for each variable of interest |  | Yes “unknown” outcomes shown in table 1 where applicable |
|  |  | (c) *Cohort study*—Summarise follow-up time (eg, average and total amount) |  | Not applicable |
| Outcome data | 15* | *Cohort study*—Report numbers of outcome events or summary measures over time | *10, 12* | *Table 3 and Table 4 and Table 5* |
|  |  | *Case-control study—*Report numbers in each exposure category, or summary measures of exposure | *-* |  |
|  |  | *Cross-sectional study—*Report numbers of outcome events or summary measures | *-* |  |
| Main results | 16 | (*a*) Give unadjusted estimates and, if applicable, confounder-adjusted estimates and their precision (eg, 95% confidence interval). Make clear which confounders were adjusted for and why they were included | 13 | Two statistical test used, risk ratio with p value. |
|  |  | (*b*) Report category boundaries when continuous variables were categorized |  | Not applicable |
|  |  | (*c*) If relevant, consider translating estimates of relative risk into absolute risk for a meaningful time period |  | Not applicable |

Continued on next page

| Other analyses | 17 | Report other analyses done—eg analyses of subgroups and interactions, and sensitivity analyses |  | Not applicable |
| --- | --- | --- | --- | --- |
| Discussion | | | | |
| Key results | 18 | Summarise key results with reference to study objectives | 14 | Discussion section reviews results and contextualises them. |
| Limitations | 19 | Discuss limitations of the study, taking into account sources of potential bias or imprecision. Discuss both direction and magnitude of any potential bias | 14 | Dedicated section on strengths and limitations |
| Interpretation | 20 | Give a cautious overall interpretation of results considering objectives, limitations, multiplicity of analyses, results from similar studies, and other relevant evidence | 13 | Discussion section reviews results and contextualises them. |
| Generalisability | 21 | Discuss the generalisability (external validity) of the study results | 14 | Compared data to previous studies. |
| Other information | |  | | |
| Funding | 22 | Give the source of funding and the role of the funders for the present study and, if applicable, for the original study on which the present article is based | 16 | Not applicable, but included conflict of interest and funding section |

*Give information separately for cases and controls in case-control studies and, if applicable, for exposed and unexposed groups in cohort and cross-sectional studies.

**Note:** An Explanation and Elaboration article discusses each checklist item and gives methodological background and published examples of transparent reporting. The STROBE checklist is best used in conjunction with this article (freely available on the Web sites of PLoS Medicine at http://www.plosmedicine.org/, Annals of Internal Medicine at http://www.annals.org/, and Epidemiology at http://www.epidem.com/). Information on the STROBE Initiative is available at www.strobe-statement.org.
